# Updating and evaluating a research best practices training course for social and behavioral research professionals

**DOI:** 10.1017/cts.2023.702

**Published:** 2023-12-27

**Authors:** Elias Samuels, Mary R. Janevic, Alexandra E. Harper, Angela K. Lyden, Gina M. Jay, Ellen Champagne, Susan L. Murphy

**Affiliations:** 1 Michigan Institute of Clinical and Health Research, University of Michigan, Ann Arbor, MI, USA; 2 Department of Health Behavior and Health Education, School of Public Health, University of Michigan, Ann Arbor, MI, USA; 3 Department of Physical Medicine and Rehabilitation, University of Michigan, Ann Arbor, MI, USA; 4 Clinical Trials Support Office, University of Michigan, Ann Arbor, MI, USA

**Keywords:** Social and behavioral research, community and stakeholder engagement, clinical and translational research, mixed methods, program evaluation

## Abstract

**Introduction:**

The clinical and translational research workforce involved in social and behavioral research (SBR) needs to keep pace with clinical research guidance and regulations. Updated information and a new module on community and stakeholder engagement were added to an existing SBR training course. This article presents evaluation findings of the updated course for the Social and Behavioral Workforce.

**Methods and Materials:**

Participants working across one university were recruited. Course completers were sent an online survey to evaluate the training. Some participants were invited to join in a focus group to discuss the application of the training to their work. We performed descriptive statistics and conducted a qualitative analysis on focus group data.

**Results:**

There were 99 participants from diverse backgrounds who completed the survey. Most reported the training was relevant to their work or that of the study teams they worked with. Almost half (46%) indicated they would work differently after participating. Respondents with community or stakeholder engaged research experience vs. those without were more likely to report that the new module was relevant to study teams they worked with (*t* = 5.61, *p* = 0.001), and that they would work differently following the training (*t* = 2.63, *p* = 0.01). Open-ended survey responses (*n* = 99) and focus group (*n* = 12) data showed how participants felt their work would be affected by the training.

**Conclusion:**

The updated course was rated highly, particularly by those whose work was related to the new course content. This course provides an up-to-date resource for the training and development for the Social and Behavioral Workforce.

## Introduction

The training needs of the clinical and translational research workforce are evolving to include new scientific areas and good clinical practices (GCP). More specialized training for the Social and Behavioral Workforce who work under the clinical and translational research umbrella is particularly needed [1–5]. Progress has been made defining the important elements of health research training and using robust evaluation methods to assess the impact of training programs on the work of scholars, trainees, and research staff [6–10]. Recent empirical research also demonstrates an emerging need to integrate new course content on community and stakeholder engagement into existing training programs [11–13]. Equipping the Social and Behavioral Workforce with knowledge about community and stakeholder engagement can facilitate collaborations with clinical and basic scientists, community partners, patients, and healthcare providers [14]. The aim of this study was to detail the updates made to a social and behavioral research training course and to evaluate the participant experience and impact of completing the course.

## Background

In 2016, the National Institutes of Health (NIH) issued a policy requiring (or recommending?) NIH-funded researchers involved in clinical trials to complete GCP training and identified various training opportunities for the workforce to meet the new requirement [15]. The original Social and Behavioral Research Best Practices course was developed in 2016 by a team at the University of Michigan (U-M) to educate clinical and translational researchers to apply GCP principles to social and behavioral research [16]. This training course, which took a median of 3.2 hours to complete, contained modules on key topics including research protocols, participant recruitment and retention, informed consent communication, confidentiality and privacy, participant safety and adverse event reporting, quality control and assurance, and research misconduct. A description of the development and evaluation of the original course is available elsewhere [16]. The NIH Office of Behavioral and Social Sciences Research included this course in online training resources offered to the health research workforce [17]. The Collaborative Institutional Training Initiative (CITI) program also offered the training course for health researchers needing an advanced refresher course in GCP [18]. At U-M, the original course was offered through the university’s learning management system (LMS), from 2016 through 2020, during which 793 individuals completed the course.

In 2021, researchers at U-M revised the GCP course to provide updated information on research regulations, practice, and community and stakeholder engagement. This update was also made in response to the need to train the Social and Behavioral Workforce in community and stakeholder engagement methods [14]. The team working on this update included U-M faculty and research staff, including those who had expertise in community-engaged research; and subject matter experts working at the NIH. The team revised the course between 2021 and 2022 and evaluated it from 2022 to 2023.

**Figure 1.**
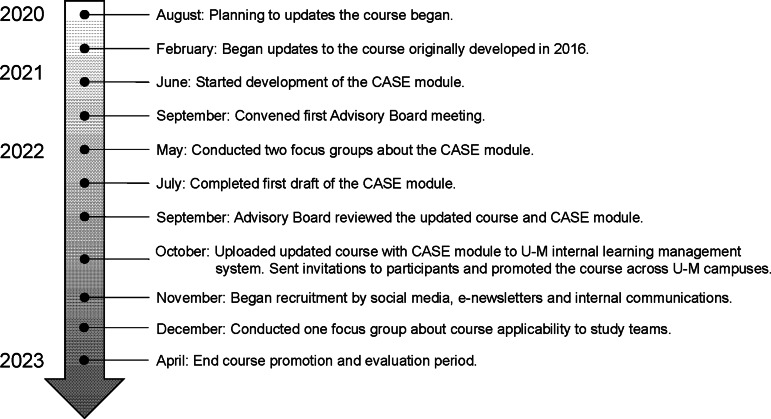
Timeline of updates made to the SBR course and of the course evaluation.

This paper describes the updates of the course and its evaluation, as illustrated by the timeline shown in Fig. 1. This course was updated to: 1) add training on regulatory changes and reporting requirements, including guidance for clinical trials, 2) incorporate features enhancing accessibility of the course, and 3) develop a new module on Community and Stakeholder Engagement (CASE). We use the term “community and stakeholder engagement” to be broadly focused on well-established methods of community engagement as well as engagement with other stakeholder groups, including participants of clinical and translational research studies. Focus groups were conducted to inform the course updates. Our team then evaluated the learners’ experiences used data extracted from the U-M LMS and from participant surveys and focus groups about impact of the course on their work. We also examined whether responses varied by learner characteristics, including past research experience, professional credentials, and demographic backgrounds including participants’ age, sex, race, and ethnicity to test our hypothesis that completing the updated training course would have a positive impact on all members of the Social and Behavioral Workforce, including those with and without community and stakeholder engagement experience.

## Materials and methods

### Updating the training course

#### Content

The U-M study team met bi-weekly or monthly starting in 2021. The first eight months of the project were dedicated to reviewing the course to identify necessary updates, drafting new course materials, and redesigning the functionality of the training modules. The team went module-by-module to determine updates needed to the course content. In general, the team wanted to ensure the recommended practices reflected updated regulatory guidance, appropriate terminology, and new developments in technology. The content changes to specific modules are outlined in Table 1. These updates included improving the quality of the knowledge checks and the exam embedded in several modules the course. For the knowledge checks and exam questions, we examined data from learners participating in the original course to determine if any questions appeared confusing or difficult to answer based on their responses. We reworded questions that appeared ambiguous and ensured that questions reflected appropriate terminology and scenarios.

**Table 1.** Updates for the social and behavioral research training course by module

Course Modules	Major Changes
Introduction	Added context to highlight skills learners need to have to conduct a research study with rigor and outlined all course module topics. Clarified roles and responsibilities of study team, such as the role of a data safety monitor in a behavioral research study Added Data Safety Monitoring Board and Advisory Board to roles Removed Office of Research Integrity to add elsewhere
Research Protocol	Rewrote the module to focus on protocol document as the foundational study document and how it informs supporting documents Reframed the module to better reflect a best practice in the video learning scenario and a more common issue that study teams might encounter Eliminated potentially confusing terms “clinical protocol,” and “IRB protocol” to the more general term of protocol document Clarified that the protocol is a living document that needs to be updated and amended as needed Removed details about protocol deviation reporting to add elsewhere Added resources for protocol document writing, such as the NIH behavioral clinical trial protocol template
Recruitment and Retention	Added information about including community advisory boards or stakeholders into developing recruitment strategies Added information about social media and online recruitment Added practical strategies in best practices regarding accommodating research participants
Informed Consent Communication	Changed knowledge check question feedback to be more accurate Updated confidentiality information and information about incentive payments Added information about e-Consent Added considerations in the consent process for people with cognitive impairments or hearing/visual disabilities
Privacy and Confidentiality	Updated knowledge check responses to remove old technology (thumb drives) and provide better explanations when a wrong answer is selected Added information about data sharing, including a data sharing plan Added information about password protection such as two factor authentication Updated images of outdated technology, like a mini cassette recorder
Participant Safety and Adverse Events	Changed knowledge check questions around the video to be more nuanced and provide more information for learners Added information about data safety and monitoring plans and study-specific adverse event reporting Added an “unanticipated problems” section Added clarifying information about classifying and reporting adverse events
Quality Control and Assurance	Revised knowledge check responses Added regulations for reporting requirements of results and importance of logging issues affecting data quality
Research Misconduct	Changed the guidance to reflect contacting Office of Research Integrity to discuss potential research misconduct instead of IRB
Conclusion	Added CASE module to course summary

#### Presentation and functionality

An instructional design company helped redesign existing training modules and the development of the new CASE module. This company redesigned videos, graphics, text, and course functionality. The course was made more accessible to learners with visual impairment. We changed how the course manual was referred to in the course. In the original course, this manual appeared in each module. We updated the manual and kept it in the resources section of the course but no longer referred to it as an interactive part of each module. We developed “key principles” to remember at the end of each module. We also added the anticipated time to complete each module. The structure of the modules, including the CASE module, was changed to accommodate the additional information. An example of the structure is provided in Table 2.

**Table 2.** Community and stakeholder engagement module structure

1	An introductory video about a hypothetical investigator who is trying to engage community members as partners.
2	Information about how researchers should understand the context for community-engaged research.
3	The history of abuses and mistrust of research participants.
4	Key definitions associated with community-engaged research.
5	Illustrative case studies demonstrating the differences between the key terms defined.
6	Information about the benefits of community engagement and the purpose and function of Community Advisory Boards.
7	A review of common challenges encountered by study teams conducting community-engaged research.
8	Practical strategies and tips to promote community engagement
9	A summary of all seven key learning objective
10	A concluding video providing resolution to the introductory video scenario
11	An embedded final exam.

#### Developing the CASE module

The new CASE module was designed to be relevant to members of the Social and Behavioral Research Workforce, whether or not they are directly involved in community-engaged or patient-centered research. However, we hypothesized that participants involved in research most relevant to the content of the CASE module would find it more impactful than those not involved in this type of research. This hypothesis is based on well-established theories of adult learning and organizational change. The theory of sensemaking hypothesizes that the ways people understand new or different information they notice is greatly affected by what information they are already familiar with [19–23]. For this reason, we hypothesize that participants with experience conducting community and stakeholder engaged research will report the course to be more relevant and impactful than those participants without such experience.

#### Subject matter expert participation

Focus group data were collected from individuals employed as faculty or researchers in eight states including Michigan, all of whom had considerable experience conducting and teaching community-engaged research. These participants had decades of expertise in academic-community partnerships, community-engaged research, health disparities, institutional review boards, research coordination, research regulation, patient engagement, patient-centered research, public health, workforce development, and underserved populations in health care. The individuals who participated in the focus groups used to inform the development of the CASE module were selected by the authors based on their subject matter expertise. These individuals were contacted by the authors and invited to share their feedback about the development of this module through a focus group that was conducted virtually.

Two focus groups were conducted in May 2022 with seven and five individuals participating, respectively. Participants were prompted to describe their view of benefits and challenges to engaging communities in social and behavioral research. This was followed by a brief presentation about the background of the new CASE training module developed by the team, the structure of which is shown in Table 2. This was followed by a review of the seven learning objectives which included, (1) describing how research can engage communities to help reduce inequities, (2) explaining the benefits of community and stakeholder participation in research, (3) showing how the history of mistreatment of research participants can affect today’s community-engaged partnerships, (4) presenting various approaches of effectively engaging communities and other stakeholders in research, (5) naming common challenges of engaging community partners and other stakeholders in research, and possible solutions, (6) describing the role and functions of a Community and Stakeholder Advisory Board across study phases, and (7) summarizing best practices that can be used to engage community partners and stakeholders in research.

Focus group participants were then asked to join in an open discussion about three key topics: 1) what participants liked about the topics addressed in the community engagement module; 2) the aspects or sections of the training course that were most important; and 3) what key information or resources were missing from the module.

Transcripts of the focus group were reviewed by the team and used to redesign the community engagement module by reframing key information and adding information and resources about community engagement to the course. For example, additional resources about community advisory boards were included, and figures depicting models of community engagement were restyled to make them easier to understand. The redesigned course was then made available to U-M employees through the university’s LMS in August 2022.

### Evaluating the updated training course

#### Participants

Data were collected from participating social and behavioral research professionals working in health research at U-M’s campuses in Ann Arbor, Dearborn and Flint, Michigan. Multiple strategies were used to recruit U-M faculty and staff working at U-M campuses to participate in the redesigned course. The participants included individuals who had completed the original iteration of the course as well as individuals who had never taken any iteration of the course before. All U-M employees who had completed the original course through the U-M LMS before 2018 were identified (*N* = 793), of whom 534 were still employed at U-M at the time of this study and who received email invitations to participate in the course and an evaluation survey administered online using Qualtrics. The course and survey invitations were personalized. In parallel, the course and survey were advertised to department chairs at U-M schools and colleges on all three campuses. Promotions were included in newsletters, social media, and special communications sent to researchers across the university from November 2022 through April 2023 by the Michigan Institute for Clinical and Health Research (MICHR).

Low participation rates in the first weeks of the study period motivated the team to use financial incentives for participant recruitment. To promote participation in the course evaluation, a $50 incentive was provided to all course completers who finished the survey. To ensure equity of treatment, participants who finished the survey before the incentive was offered were also contacted and offered $50 for their involvement.

#### Methods

Another focus group was conducted in December 2022 to discuss participants’ understanding of how they could utilize the training course in practice, particularly within their study teams planning or implementing Community-Based Participatory Research (CBPR) approaches to research, patient-oriented research, and other community-engaged research practices [24]. Participants were recruited from U-M staff who volunteered for a standing working group at MICHR to advance workforce development initiatives. This working group consisted of 12 health research staff employed in a variety of schools, colleges, and administrative units at U-M’s Ann Arbor campus who had all taken the updated training and participated in the evaluation survey. We asked a series of questions about the participants’ experiences of attending the training course as well as the potential of utilizing the lessons learned to advance the professional development of their study teams conducting social and behavioral research. A recording of the focus group was transcribed and analyzed.

#### Evaluation outcome measures

Training outcomes were measured via the participants’ experiences, context-based learning, and behaviors, as well as their perceptions of the impact of the training overall [25–26]. These outcomes included measures of: 1) the relevance of the overall training, 2) the relevance of the CASE module specifically, 3) whether participants’ colleagues care about the issues the training addresses, 4) the need for this training among the health research workforce, and 5) the intent of participants to work differently as a result of the training course. As a measure of their satisfaction with the course, we asked if participants would recommend the course to their colleagues and/or to partnering organizations and groups. All training outcomes were measured on the same Likert scale of agreement, in which 1 = Strongly disagree, 2 = Somewhat disagree, 3 = Neither disagree nor agree, 4 = Somewhat agree, and 5 = Strongly agree. Participants were asked in open-ended survey questions to describe ways in which the course was useful and how they could use the resources provided in the course individually and within their study teams.

#### Survey data analysis

Participant data were extracted from U-M’s LMS indicating course completion, the amount of time taken to complete each module, and performance on the embedded exam. These data were aggregated and compared between those participants who participated in the survey and those who did not. Descriptive statistics were used to compare different groups of participants’ experience in the updated course. Responses to open-ended questions were analyzed qualitatively to identify representative testimonials of impact.

Survey data were collected on participants demographic characteristics and professional background, including their roles, certifications, degrees, workplace settings, experience with social and behavioral research, and work with community-engaged or patient-centered research. We examined how participants’ perceptions of the entire course to their work varies across participants with and without experience conducting community-engaged research. Participants with research experience relevant to community and stakeholder engagement were identified as those individuals who indicated either that (a) they work on a study team conducting community-engaged or patient-centered research or (b) they work on research studies at a community site as a paid worker or volunteer. ANOVAs, t-tests, and ranked correlations were used to identify statistically significant differences between participant groups based on their past experience conducting community and stakeholder-engaged research.

#### Focus group data analysis

The semi-structured interview protocol used for the 1-hour focus group was followed by analysis of focus group recordings and notes. Grounded theory was used to analyze the focus group results for themes concerning the use of the training resources within study teams [27]. The first author generated resultant codes which were reviewed by the study team which identified main themes and determined that saturation was reached. The results were used to inform to their conclusions about the evaluation of the course.

## Results

A total of 187 individuals completed course between August 2022 and April 2023. Most individuals completed the training course in approximately four hours (Mean = 4.1 hours, SD = 3.4 hours, Median 3.5 hours). On average, participants answered 89% of the exam questions correctly (SD = 3.4%). Every individual who completed the course was sent an invitation to take the survey of which 99 (53%) answered at least one question. The vast majority of participants (*N* = 93, 93.9%) completed over 90% of the survey form. The survey participants completed the course in roughly the same amount of time and with a similar score as the course participants considered as a whole (Mean course duration = 4.1 hours, SD = 3.2 hours, Median = 3.5 hours; Mean exam performance = 88.9% correct, SD = 3.4%).

### Survey results

#### Participant characteristics

Of the survey participants, most identified as being female (79%) and White (69%). On average, the respondents were 43 years of age (SD = 13.0). As shown in Table 3, a substantive proportion self-identified as members of underrepresented minority groups.

**Table 3.** Survey Participant Demographics

Demographic Categories *(Summative notes below)*	N	%
Total Respondents	99	100%
Race		
White	69	69.7%
Asian	12	12.1%
More than one Race	5	5.1%
Black or African American	3	3.0%
Other	1	1.0%
Ethnicity		
Not Hispanic or Latino	81	81.8%
Hispanic or Latino	9	9.1%
Sex		
Female	79	79.8%
Male	13	13.1%
Gender		
Female	76	76.8%
Male	13	13.1%
Genderqueer, gender nonconforming, neither exclusively male nor female	3	3.0%
Underrepresented Populations in the Health Research Workforce		
Individuals with disabilities	23	23.2%
Women from underrepresented backgrounds at the graduate level and beyond in doctorate-granting research institutions at senior and other faculty levels in most biomedical-relevant disciplines.	16	16.2%
Individuals from other defined disadvantaged backgrounds.	11	11.1%
Individuals from racial and ethnic groups that have been shown to be underrepresented in health-related sciences on a national basis.	9	9.1%

*Notes:*
Respondents were allowed to choose as many race categories as applicable. 90 individuals selected 95 categories, including the other racial groups they specified for themselves. Respondents who selected more than one race, including ‘Other’ selections, were recoded as “More than one race.” 90 individuals identifying themselves as either one of two ethnic categories. 92 individuals identifying themselves as either one of two sex categories. 92 individuals identifying themselves as either one of three gender categories. Respondents did not choose three additional categories, including 1) Transgender man/ trans man/ female-to-male (FTM), 2) Transgender women/ trans woman/male-to-female (MTF), or 3) Additional gender category. Respondents were allowed to choose as many underrepresented minority categories as applicable. 41 individuals selected 59 categories. The definitions of each category are detailed in the NIH’s notice of institutes’ interest in diversity [28].

Of note, 41% of participants considered themselves to be underrepresented in the extramural scientific workforce, as defined by the NIH [28]. Many identified as belonging to racial and ethnic groups that have been shown to be underrepresented in health-related sciences on a national basis (9%) or were from disadvantaged backgrounds (11%). A notable proportion identified as having a disability (23%). Many (16%) of the participants identified as women from the above backgrounds working at the graduate level and beyond in doctorate-granting research institutions or working at senior and other faculty levels in biomedical-relevant disciplines.

Participants’ professional experiences were also diverse (Table 4). While two-thirds were research staff and administrators (66%), a substantial proportion identified as postsecondary students (16%). A smaller proportion of respondents were faculty (9% university faculty and 4% clinicians). Most (91%) had earned postsecondary degrees, with many possessing a Master’s degree (41%) or Doctorate (16%). Some (12%) reported achieving certification by the Society of Clinical Research Associates (SOCRA) (5%), the Association of Clinical Research Professionals (ACRP) (2%), or another professional certification (5%). Of the 99 individuals who filled responded to the evaluation survey, 16 (16%) had completed the original version of the course in the past as well as the updated version of the course evaluated here.

**Table 4.** Survey participant professional experiences

Professional Categories *(Summative notes below)*	N	%
Role		
Research Coordinators, Project Managers, Associates & Area Specialists	36	36.4%
Research Assistants & Technicians	32	32.3%
Graduate & Undergraduate Students	16	16.2%
Faculty	9	9.1%
Clinician	4	4.0%
Certifications		
SOCRA	5	5.1%
Other	5	5.1%
ACRP	2	2.0%
Higher Education Degrees		
Bachelor’s Degree (e.g., BA, BS, BSN)	53	53.5%
Master’s Degree (e.g., MA, MS, MHA)	41	41.4%
Doctorate (e.g., MD, PhD, DrPH)	16	16.2%
Associate’s Degree (e.g., AA, AS, ADN)	4	4.0%
Other Professional Degree	3	3.0%
Workplace Setting		
Research University	68	68.7%
Academic Medical Center	42	42.4%
Community Organizations or Settings	12	12.1%
Governmental Agency	3	3.0%
Other	2	2.0%
Other Research Experience		
Work on clinical, translational, or social and behavior research studies.	69	69.7%
Work on a study team conducting community engaged or patient centered research.	43	43.4%
Other work.	6	6.1%
Work affiliated with a CTSA-funded institution	5	5.1%
Work on research studies at a community site as a paid worker or volunteer.	4	4.0%

*Notes:*
Respondents were allowed to choose as many roles as applicable. 93 individuals selected 123 roles, including other self-specified roles. Respondents were allowed to choose as many certification categories as applicable. 12 individuals selected 12 categories. Other specified options all included references to professional credentials or training certifications. Respondents were allowed to choose as many credential categories as applicable. 91 individuals selected 117 categories. Other Professional degrees include: 1) Current MA student, 2) Current OTD student, 3) Graduate certificate. Respondents were allowed to choose as many work location categories as applicable. 93 individuals selected 128 categories. Other specified locations included a reference to outpatient clinics. Respondents were allowed to choose as many current work categories as applicable. 91 individuals selected 128 categories. Other specified options include references to different types of research work, including bioinformatics research, cancer research, qualitative research, and outcomes research.

Participants were asked to identify their work settings and the types of research in which they are engaged. Most reported that their work settings included a research university (68%) or an academic medical center (42%). Also, 12% reported working in community-based organizations or community settings. Few reported working at governmental agencies (3%), and 2% also reported “other” work settings. Notably, 43% of participants reported having worked on a study team conducting community-engaged or patient-centered research. Most (69%) worked on clinical, translational, or social and behavioral research studies. On average, the participants had worked on social and behavioral research studies for over 5 years (Mean = 5.9, SD = 7.3).

#### Survey evaluation outcomes

Most participants strongly agreed the course was relevant to their own work (69%) or that it was relevant to the study teams with whom they work (63%). On average participants strongly agreed to both outcome statements (Mean = 4.6, SD = 0.8 & Mean = 4.6, SD = 0.6, respectively). Participants also agreed that the CASE module was relevant to study teams with whom they work (Mean = 4.2, SD = 0.9).

The participants who indicated that the CASE module was relevant to their work were asked to explain their reasoning in an open-ended question. They responded with examples regarding their past and future work, ranging from applications to their understanding of CBPR, formation of community advisory boards, participant recruitment, and participant interactions involving vulnerable populations. Examples of respondents’ comments of the relevance of the training course to their past and future work follow, respectively:

*“We interact and collaborate with community members and organizations to help guide and support our research study. In order to utilize the best practices available, we really count on our community advisory board members and focus group participants being as engaged in the research as possible. So, the strategies for community and stakeholder engagement were very useful for me.”*

*“I find that [the CASE module] is particularly relevant for the consideration of brainstorming and devising new, relevant, and interesting research questions by utilizing the resources and knowledgebase of the community. Additionally, the module provided useful ideas for how to engage and involve the community/stakeholders to enrich various aspects of research projects I may be working on in the future.”*

The survey participants also strongly agreed that all members of the clinical and translational research workforce need this course to conduct social and behavioral research (Mean = 4.6, SD = 0.7). Only three respondents (3%) disagreed with this statement and one neither agreed nor disagreed. When asked if they would work differently as a result of having received the training, only 7 individuals disagreed, 42 respondents neither agreed nor disagreed, and just under half (46%) agreed that the course would cause them to work differently in the future (Mean = 3.5, SD = 0.8).

Those who indicated that they would work differently as a result of the training course were asked to describe how they expected to do so in an open-ended question. They responded with examples regarding their current and future behavior, ranging from their adherence to GCP, use of best practices for patient interactions, increased awareness of the proper use of research protocols, and ability to generate new health research questions. Quotations of respondents’ examples of the application of the course to their current and future work follow, respectively:

*“Many of the issues discussed in the training are relevant to the work of our research team, and we often discuss these issues. The training made certain aspects of our work more salient for me and had already led me to produce and share materials related specifically to our data security.”*

*“I will be even more conscious that the communities I interact with are experts in their own experience and [will] keep in mind that I remain a neutral party in conversation and emphasize the participants autonomy.”*

The results of this course were also evaluated using two measures regarding the intention of participants to recommend the training to their colleagues and to the organizations or groups with whom they work [29–30]. Substantial majorities agreed that they would recommend this course to their colleagues (80%) and to the organizations and groups with whom they work (76%). On average, they agreed that they would recommend their training experience to both the individuals and sets of people that they worked with (Mean = 4.4, SD = 0.8; Mean = 4.2, SD = 0.9, respectively).

Those who agreed that they would recommend the course to others were asked why they expected to do so in an open-ended question. Responses typically referred to the fundamental value of the training course, both as an introduction for those new to research and as a refresher for those with considerable experience. Examples of respondents’ reasoning behind their intention to recommend the training to individuals and to organizations or groups they work with follow, respectively:

*“I will recommend this training to every new person working in research. This training is fundamental and every person who works on research needs to be trained on these topics. … There are new employees who do not have a research background and need the basic/fundamental training in order to perform well in their job.”*

*“I think this training would be really helpful for community orgs that are embarking on training with [the University]. Too often they really aren't aware of all that goes into research and making sure someone is giving full consent.”*

#### Association between participant backgrounds and evaluation outcomes

Individuals with research experience relevant to community and stakeholder engagement were more likely to report that the CASE module was relevant to the study teams with whom they work than those participants without such research experience (*t* = 5.6, *p* = 0.001, *µ* = 4.50_with relevant research experience_, *µ* = 3.89_without relevant research experience_). These individuals were also more likely to indicate that they would work differently as a result of the training course considered overall (*t* = 2.6, *p* = 0.01, *µ* = 3.73_with relevant research experience_, *µ* = 3.29_without relevant research experience_).

A small positive ranked correlation was found between the number of years of experience participants reported working on social or behavioral research and the likelihood that they would agree the training course was relevant to the study teams with whom they work (*N* = 91, Spearman’s rho = 0.23, *p* = 0.03). No other significant differences were found based on participant demographics. The demographics categories that were tested included participants’ race, ethnicity, sex, gender, and underrepresented status in the clinical and translational research workforce. We did not hypothesize that there would be significant differences between participant demographic groups. Instead, these analyses reflect the importance of acknowledging the inherent diversity of participants’ learning experiences [26,31].

### Focus group results

There were three primary conclusions made by the participants in the focus group about the application of the course to the work of the clinical and translational research workforce. Participants noted that the impact of the course could be enhanced by focusing on content that is most relevant to participants or teams. For example, they discussed the possibility of using the CASE module content and resources as a training tool with study teams or other individuals that were conducting community-engaged research. They also articulated their belief that the training course can provide social and behavioral researchers with a common understanding of their work. Third, they concluded the course would be most relevant to those who regularly engaged in social and behavioral research.

Table 5 provides the counts of the most frequently used codes applied to the focus group transcript. The representative quotes in this table also demonstrate how participants imagined the interplay between similar colleagues’ potential participation in the course and their similar reactions to the experience. This result is best illustrated by the following representative quote:

**Table 5.** Top codes for focus group on participants application of the course to practice

Selective Codes	Code Count	Representative Quote
The course provides the workforce with a common understanding of their work.	12	“I think [the training] would support what the faculty and what I have been trying to standardize across the center in terms of protocols. And it would give from project managers down to data collection staff or phlebotomist, or whoever’s on our team, an appreciation for the role. … The Protocol, and the manual of operations, and the standard operating procedures - why those are all important this time. I think that that’s really important to have that additional support.”
The course is important to those who are regularly engaged in the research the course describes.	9	“One of the things that I appreciate about this entire [training] is that it is not something that is overly focused on FDA compliant trials. The fact that this is really social behavioral research and is focused on the kind of work that might teams generally do already makes it far more relevant and impactful than something where I will see my low-level RAs eyes start to glaze over because they don't do that kind case report forms where we have an external sponsor where we're sending drug information to the FDA. I mean that’s what really makes it salient is the fact that it is for the kind of work we do.”
The impact of the course could be enhanced by focusing on the content that is most important to similar participants or teams	8	“Going back to that community of practice type thing… Going back and having a [training] resource for other study team members that you can call and be like, “hey you want to come [and] watch our study, visit, or observe, or walk through our process with me?” …’ But just having that built-in community and resource, I think, would be really, really beneficial.”

*“Going back to that community of practice type thing… Having a [training] resource for other study team members that you can call and be like, “hey you want to come [and] watch our study, visit, or observe, or walk through our process with me?” versus having to ask a PI to say, “Hey, can you contact so and so? [Or] do you know anybody that might be able to do that?” But just having that built-in community and resource, I think, would be really, really beneficial.”*

The representative quotes in Table 5 also show how participants understood the alignment of their training experience with their work and how participants’ training experience reinforced their prior beliefs about their work. The representative quotes below respectively characterize both aspects of participants’ training experience:

*“One of the things that I appreciate about this entire [training] is that it is not something that is overly focused on FDA compliant trials. The fact that this is really social behavioral research and is focused on the kind of work that [study] teams generally do already makes it far more relevant and impactful …. I mean that’s what really makes it salient is the fact that it is for the kind of work we do.”*

*“I think [the training] would support what the faculty and what I have been trying to standardize across the center in terms of protocols. And it would give from project managers down to data collection staff or phlebotomist, or whoever’s on our team, an appreciation for the role. … I think that that’s really important to have that additional support.”*

## Discussion

This work details (1) the approach our team took to updating a training course for best practices in social and behavioral research and (2) the results of the evaluation of the participants’ experiences and impact of the knowledge on their work. This approach enabled us to illuminate connections between the professional development of the Social and Behavioral Workforce and the contributions of this workforce to the advancement of clinical and translational research [32–35]. The results of our study make it reasonable to claim that participants outside our university system will find the new training content relevant to their shared work. The feasibility and effectiveness of the process used to update this course suggest that the incorporation of best practices for community and stakeholder engagement into existing educational opportunities may be one strategy to prepare the workforce to conduct social and behavioral research in partnership with communities.

The results of this study suggest this training module is broadly applicable to this research workforce. Community and stakeholder engagement in social and behavioral research advances translational science by involving typically underrepresented populations in research studies [36–38]. As such, standardizing participation in this course can contribute to the ability of the workforce to accelerate the translation of discoveries into interventions and policies that improve the health of all people. Future research should focus on the facilitators and barriers to the long-term impact of this and similar trainings designed for the clinical and translational research workforce. Improving the quality of the training creates more opportunity to professionalize the clinical and translational research workforce [39–40]. The need for more impactful community-engaged health research depends on the capacity of these interdependent workforces to be efficiently trained and adequately prepared to conduct community-engaged research that is meaningful to researchers and communities [41–43].

The results of the study further suggest that this updated training course was acceptable and valued by members of the Social and Behavioral Workforce, including those with professional experience in community and stakeholder engagement. In this respect, the updated course has the potential to contribute to the engagement of underrepresented minorities across this workforce. Future research may consider evaluating the impact of such training on study teams’ engagement with and inclusion of minority groups in their research studies [41–43]. Moreover, the presence and support of underrepresented minorities working in academic medical centers helps to guarantee diverse research and mentorship experiences for junior investigators, helping to enhance critical representation in the workforce [44–45].

Our process and evaluation also demonstrate the feasibility of updating critical training resources for the Social and Behavioral Workforce. Although this occurred only at the University of Michigan, having experience in updating and improving training opportunities for researchers can enhance the university’s capacity to anticipate and adapt to advances in clinical and translational research and sudden environmental changes, such as the onset of the COVID-19 pandemic [46–47]. The improvement of this training course also promotes institutional buy-in into research topics of importance to the broader public, notably including communities impacted by health disparities [48–49].

## Limitations

This work has several limitations that should be kept in mind. We updated this training course to be relevant to the health research workforce and administered it within only one university system. The low response rate to the survey is comparable to similar online surveys administered via email [50]. The use of a financial incentive may have had a disproportional impact on the willingness to participate, although $50 to complete a 4-hour training course and evaluation seems appropriate. The incentive may have also systematically biased responses. The generalizability of this study is limited to one large research university and the participating research professionals may differ from those working in other settings in ways that might affect the impact of the training experience. Moreover, the scope of this study could not be extended to include an evaluation of the long-term impact of social and behavioral research training course on the work of the research workforce. Therefore, our conclusions are limited to the short-term impacts.

## Conclusion

This work presents evidence that this revised social and behavioral research training course is highly relevant to growing proportions of the clinical and translational research workforce involved in community and stakeholder engagement. More broadly, this course provides a standard training, which applies to all members of the clinical and translational research workforce engaged in social and behavioral research. The approach used to update and evaluate this training was effective and is reproduceable.
